# Physiological basis for low-temperature survival and storage of quiescent larvae of the fruit fly *Drosophila melanogaster*

**DOI:** 10.1038/srep32346

**Published:** 2016-08-30

**Authors:** Vladimír Koštál, Jaroslava Korbelová, Tomáš Štětina, Rodolphe Poupardin, Hervé Colinet, Helena Zahradníčková, Iva Opekarová, Martin Moos, Petr Šimek

**Affiliations:** 1 Institute of Entomology, Biology Centre CAS, Branišovská 31, 37005 České Budějovice, Czech Republic; 2 Faculty of Science, University of South Bohemia, Branišovská 31, 37005 České Budějovice, Czech Republic; 3 Institut für Populationsgenetik, Vetmeduni Vienna, Vienna, Austria; 4 Université de Rennes 1, UMR CNRS 6553 ECOBIO, 263 Avenue du Général-Leclerc, 35042 Rennes, France

## Abstract

The cryopreservation techniques proposed for embryos of the fruit fly *Drosophila melanogaster* are not yet ready for practical use. Alternative methods for long-term storage of *D. melanogaster* strains, although urgently needed, do not exist. Herein, we describe a narrow interval of low temperatures under which the larvae of *D. melanogaster* can be stored in quiescence for up to two months. The development of larvae was arrested at the pre-wandering stage under fluctuating thermal regime (FTR), which simultaneously resulted in diminishing the accumulation of indirect chill injuries. Our physiological, metabolomic, and transcriptomic analyses revealed that compared to larvae stored at constant low temperatures, the larvae stored under FTR conditions were able to decrease the rates of depletion of energy substrates, exploited brief warm episodes of FTR for homeostatic control of metabolite levels, and more efficiently exerted protection against oxidative damage.

The potential to store insects for a long term at low temperatures would procure considerable benefits. Long-term storage could supplement, or even replace, tedious and expensive continuous rearing practices currently used in mass rearing facilities that produce insects for pest management purposes, or in large *Drosophila* centres that preserve valuable genetic lines and mutant strains. Two basic strategies for low-temperature insect storage are known: (i) cryopreservation of embryos at cryogenic temperatures, most often in liquid nitrogen at −196 °C^1^, and (ii) long-term storage at temperatures below the threshold for development, which is typically applicable for insects in diapause^2^. The methods for long-term storage of fruit flies (*Drosophila melanogaster*) although urgently desired, currently produce unsatisfactory results. Despite the growing list of successfully cryopreserved insect embryos in liquid nitrogen^1^, the attempts to cryopreserve embryos of *D. melanogaster* have been only partially successful in the past^3^,^4^. The theoretical possibility of cryogenic technique use for the conservation of complex tissues or whole organisms appeared mostly unsuccessful or very problematic in practice^5^,^6^. We previously described a protocol for cryopreservation of larvae of another drosophilid fly, *Chymomyza costata*^7^. Thereafter, in an attempt to adapt this protocol for *D. melanogaster*, we could ensure survival of fruit fly larvae after conversion of the freezable fraction of body water into ice (freeze tolerance), yet such larvae had no capacity to survive in liquid nitrogen^8^. Here, we extend our previous effort, and analyse whether the larval stage possess some capacity for long term storage at moderately low temperatures.

A strategy of long-term storage at temperatures slightly below the threshold for development, exploiting the natural features of insect diapause, presents itself as a useful alternative to cryopreservation in many insects^1^. However, *D. melanogaster* adults show very weak capacity to enter reproductive diapause^9^,^10^, and larvae possess absolutely no capacity for diapause. *D. melanogaster* is a fly of tropical origin, its larval stage is evolutionarily adapted for rapid growth and development under warm conditions, and all ontogenetic stages are highly susceptible to cold^11^,^12^. In insect species that lack diapause, the potential for practical long-term storage at low temperatures could be limited unless the host of problems linked to development of indirect chill injury is solved. The causes of indirect chill injury are insufficiently understood but likely involve a complex of detrimental changes: metabolic disorder, oxidative stress, depletion of free chemical energy, and disturbance of ionic and osmotic homeostasis^13^,^14^,^15^,^16^,^17^.

The first goal of our study was to determine the temperature conditions that are sufficiently cold to halt the developmental processes (inducing quiescence), but warm enough to avoid/slow down accumulation of indirect chill injury. Based on previous reports^18^,^19^ and our previous results^11^, we focused on constant low temperatures (CLTs) in the range from +3 to +9 °C and assessed the time limits of larval storability. Next, we attempted to extend the storage time by applying fluctuating thermal regimes (FTRs). In FTRs, relatively long periods of cold are alternated, most often on a daily basis, with relatively short periods at high/optimal temperature. In various insects, a brief exposure to high temperature allows repair of chill injury accrued during preceding cold periods and, consequently, extends storage at low temperatures (for a recent review, see ref. 20). Using a combination of low temperature-induced quiescence and FTR-based suppression of chill injuries, we were able to store viable larvae for up to 2 months. Next, we conducted metabolomic and transcriptomic analyses of CLT- and FTR-exposed larvae in order to describe the physiological basis of long-term survival of the chill susceptible larvae in quiescence. We identified developmental failures, depletion of energy substrates, loss of metabolite homeostasis and oxidative damage as potential mechanisms responsible for accumulation of indirect chill injury, which sets physiological limits on *D. melanogaster* low-temperature storability.

## Results and Discussion

### *D. melanogaster* larvae can be stored in quiescence for up to 2 months

**Figure 1:**
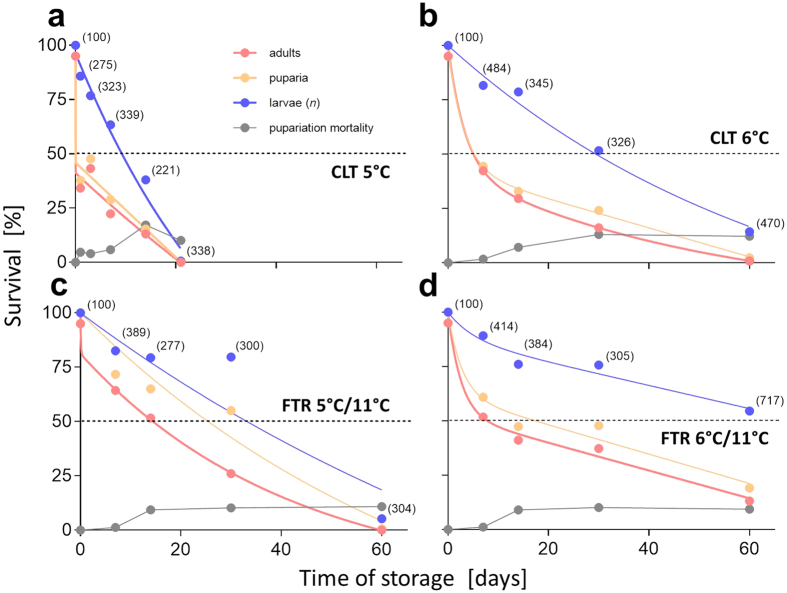
Survival under selected CLT and FTR regimes. (**a–d**) Survival of quiescent larvae *of Drosophila melanogaster* stored at two constant low temperatures (CLTs) of 5 °C (**a**) and 6 °C (**b**), and two fluctuating thermal regimes (FTRs) of 5 °C/11 °C (**c**) and 6 °C/11 °C (**d**).Three different levels of survival were scored: live larvae (blue circles, larvae showing spontaneous movements); puparia (orange circles, formation of morphologically normal puparium); and adults (red circles, eclosion of morphologically normal adult). In addition, we counted numbers of malformed puparia on the wall of the glass tube (grey circles) and scored them as ‘pupariation mortality.’ Numbers within parentheses are the total number of all individuals (*n*, including dead larvae) recovered at each time. The two-phase exponential decay curves (Prism 6.0, GraphPad, San Diego, CA, USA) were fit to survival data with a goodness of fit, R^2^: (**a**) 0.9778, larvae; 0.9800, puparia; 0.9795, adults; (**b**) 0.9871, larvae; 0.9940, puparia; 0.9952, adults; (**c**) 0.8406, larvae; 0.9219, puparia; 0.951, adults; (**c**) 0.9544, larvae; 0.9682, puparia; 0.9863, adults.

We assayed survival and storability of fully grown 3rd instar larvae of *D. melanogaster* at different thermal regimes for various periods ranging from 1 d to 60 d. Five constant low temperatures (CLTs: 3 °C, 5 °C, 6 °C, 7 °C, and 9 °C) and two fluctuating thermal regimes (FTRs: 5 °C/11 °C and 6 °C/11 °C; for more explanations, see Methods and Fig. S1a) were assessed. Upon transfer to low temperatures, larvae halted ontogeny at the pre-wandering stage and entered into quiescence. The ‘temperature-window’ for successful quiescence is theoretically delimited by the temperature of the upper limit of cold injury zone (ULCIZ) from below^21^ and the temperature of the lower developmental threshold (LDT) from above^22^. Although diapausing insects are often able to shift their ULCIZ to deep sub-zero temperatures (even to −196 °C in extreme cases^7^), the temperature-window for quiescence in *D. melanogaster* larvae is relatively narrow. It spans approximately from 6 °C (ULCIZ^11^) to 10 °C (LDT^18^,^19^). We confirmed this narrow quiescence window by observing the rapid occurrence of chilling injury and mortality in larvae at CLTs of 3 °C (Fig. S1b) and 5 °C (Fig. 1a). Conversely, most larvae continued developing, wandered, and pupariated, but were not able to proceed further in development and died at CLT 9 °C, (Fig. S1d) (‘pupariation mortality,’ see grey lines in survival diagrams). At CLTs 6 °C and 7 °C, *i.e.* at optimum temperatures for quiescence, larval survival was relatively high and small proportions of larvae (up to 13%) succumbed to pupariation mortality. The survivor larvae, however, showed impaired capacities to form puparia and emerge as fit adults. Thus, only 0.9% and 2.1%, adults emerged after 60 d-storage at CLT 6 °C (Fig. 1b) and CLT 7 °C (Fig. S1c), respectively.

Application of FTR protocol significantly improved survival at low temperatures. The positive effect of FTR was especially apparent in the experiment comparing survival at CLT 5 °C (Fig. 1a) vs. FTR 5 °C/11 °C (Fig. 1c). The highest long-term survival was recorded at FTR 6 °C/11 °C where 54.5% larvae were still alive after 60-d-long storage and 13.1% healthy adults emerged (Fig. 1d). The general rationale used to explain the positive effect of FTR on insect survival at low temperature is based on the assumption that brief, periodic exposures to high temperature allow repair of indirect chill injury accrued during preceding cold periods^20^. During warm episodes, insects could re-establish the ion balance that was partially lost during preceding chilling episodes^23^. In addition, the insects probably can exploit the warm episodes for: repair/degradation of damaged proteins via heat shock protein-assisted processing^24^,^25^; replenishment of potentially depleted ATP^26^; management of oxidative damage^27^; or synthesis of cryoprotectants that would be useful in subsequent cold spells (for detailed discussion, see ref. 20). We will discuss some of these mechanisms later.

We observed (Koštál *et al.*, unpublished observations) that a small fraction of larvae survived at FTR 6 °C/11 °C even after 3 months, but these larvae were not able to metamorphose into adults. We also observed that increasing the temperature of warm episodes of FTR by just a single degree C (from 11 °C to 12 °C) or extending the duration of the warm episode of FTR by just 2 h (from 4 h to 6 h) allowed slow continuation of larval development resulting in high pupariation mortality (Koštál *et al.*, unpublished observations). Practical applicability of the FTR 6 °C/11 °C storage protocol to other strains (other than Oregon R that was used in this study) or mutant strains of *D. melanogaster* is not straightforward, but would require careful assessment.

### Physiological limits of low temperature storability in *D. melanogaster* larvae

The insect cold tolerance literature recognizes three basic types of cold-associated injury (for review see ref. 28): *freezing* of body water resulting in cellular dehydration, osmotic concentration of solutes, and mechanical damage to cells^29^; *direct chilling* injury upon exposure to severe cold shock, which causes dissociation of multimeric proteins, protein denaturation, and membrane lipid phase transitions^30^,^31^,^32^; and *indirect chilling* injury upon chronic exposures to relatively mild low temperatures, which causes gradual accumulation of various metabolic disorders including oxidative stress, depletion of energy substrates and/or ATP, and disturbance of ionic and osmotic homeostasis^13^,^14^,^15^,^16^,^17^. Our analysis focused on indirect chilling injuries as we exposed the larvae to temperatures slightly *above* the ULCIZ.

The survival data were best-fitted using two-phase exponential decay curves (Fig. 1b–d and Fig. S1c). The first phase of fast decay of survival was observed in both CLT and FTR conditions during the early part of storage (approximately 1–2 weeks). We speculated that this high initial mortality could be linked to developmental failures in larvae that were out of the optimal ontogenetic stage for entry into quiescence (too early or too late in development) when being transferred to storage conditions. We defined the developmental failure as an inability to proceed correctly through the specific ontogenetic stage when the blockade of morphogenetic processes was incomplete during quiescence. The developmental failure was most clearly expressed in our experiments at constant 9 °C, when all larvae proceeded successfully to wandering and pupariation stages but were not able to form and proceed through the pupal stage. Later during storage, the rate of decrease in survival was much slower (second, slow decay phase). This meant that the larvae that managed to arrest their ontogeny at an optimal stage for quiescence were able to survive until a combination of accumulated indirect chill injuries killed them. The nature of chill injuries will be discussed next.

### Alterations in metabolomic profile linked to entry into quiescence

**Figure 2:**
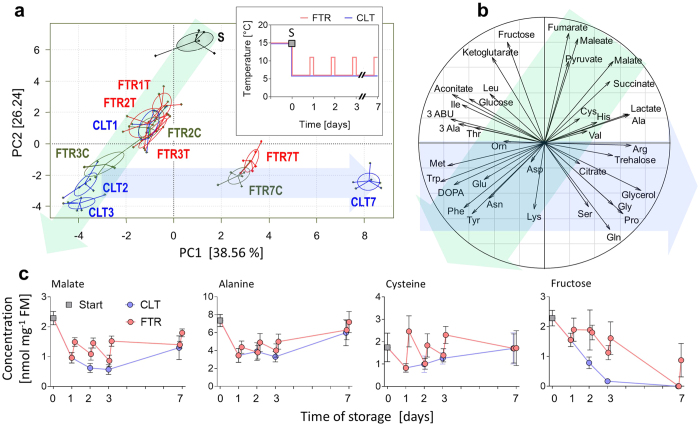
Cold-induced changes in metabolite composition. (**a**) The plot of PC1 and PC2 derived from principal component analysis shows clustering of treatments according to metabolite global composition analysed in quiescent larvae of *Drosophila melanogaster* stored at CLT 6 °C and FTR 6 °C/11 °C for 7 days. The inset schematically depicts CLT and FTR thermal regimes (for more information, see Fig. S1a). Six replicates of each treatment are linked to a common centroid by line segments; the ovals represent 95% confidence intervals around the centroid. (**b**) The projection of individual metabolites on the correlation circle helps to identify major drivers in cold-induced alteration of metabolite composition during the first 3 days (green arrow) and later (blue arrow). (**c**) examples of cold-induced concentration changes in four metabolites. For complete metabolite dataset, see Table S1.

Our metabolomic analysis revealed variations in the levels of 37 different metabolites (see complete list in Table S1) linked to storage at CLT 6 °C (will be abbreviated as CLT in next text) and FTR 6 °C/11 °C (will be abbreviated as FTR in next text). We subjected these data to between-class PCA analysis, which clustered the treatments and found statistically significant differences between them (Monte Carlo test, P < 0.001). The plot of PC1 and PC2 components, collectively accounting for 64.80% of total inertia, is presented in Fig. 2a. Exposure to 6 °C for a period as short as 18 h exerted a strong influence on metabolite composition, as witnessed by clear separation of the treatment Start from the treatment CLT1. The PCA analysis further revealed that the metabolomic response occurred in two phases. During the first phase (from Start to day 3, green arrow in Fig. 2a), the clusters of all treatments gradually moved along the PC1 and PC2 axes in negative directions. According to the projection of individual metabolites on the correlation circle (Fig. 2b), this gradual shift was driven mainly by increasing concentrations of some aromatic amino acids (phenylalanine, tyrosine, tryptophan, DOPA) and of methionine, whereas the concentrations of most organic acids (except citrate and aconitate), and fructose and alanine, decreased. The shift proceeded ‘faster’ under CLT than under FTR conditions. That was because numerous metabolites showed characteristic staircase-like trends under FTR conditions, where the concentrations were driven to opposite directions during the cold and warm episodes (see four examples in Fig. 2c). These results indicated that loss of initial metabolite homeostasis proceeded faster under CLT than under FTR. Collectively, the first phase was characterized by metabolite changes suggesting a slowdown of intermediary metabolism, including glycolysis and TCA turnover: (i) glucose slightly decreased and fructose was rapidly depleted despite that very high concentrations of both sugars were present in the larval diet^11^. These results indicated that food intake/digestion was severely compromised; (ii) pyruvate, the end-product of glycolysis, decreased and two end-products of fermentation, lactate and alanine, decreased as well; (iii) two upstream intermediates of TCA, citrate and aconitate, increased, whereas the others decreased (ketoglutarate, succinate, fumarate, and malate) suggesting a blockade of TCA at the locus of isocitrate dehydrogenase, which converts isocitrate to ketoglutarate.

The samples taken on day 7 of exposure shifted far along the PC2 axis in the positive direction. Again, the direction of this shift was similar in CLT and FTR treatments, whereas the distance shifted was longer in CLT than in FTR. During this second phase (blue arrows in Fig. 2a,b), metabolic suppression probably continued, but the metabolite composition partially recovered (the concentrations of TCA intermediates showed a trend toward initial conditions) and/or new homeostatic conditions were established. Under the new homeostatic conditions, metabolism probably shifted toward higher exploitation of proteins and lipids as energy substrates: (i) the concentrations of trehalose, a principle sugar in insect circulation^33^, were maintained high and almost constant; (ii) the breakdown of proteins was indicated by increasing concentrations of free amino acids. For instance, glutamine concentrations increased from 10.4 nmol mg^−1^ FM at Start to 29.7 nmol mg^−1^ FM (day 7 of CLT) or 34.8 nmol mg^−1^ FM (day 7 of FTR). Glutamine may serve as a sink and deposit of amino-groups released from other amino acids during their degradation; and (iii) the lipid breakdown was indicated by increasing glycerol concentrations.

The changes in metabolite profiles were broadly similar at CLT and FTR. This similarity probably reflects the fact that temperatures were favourable for survival (above ULCIZ) in both treatments. Nevertheless, larvae survived longer at FTR than at CLT and our metabolomics analysis confirmed that the warm episodes of FTR may generally serve the purpose of re-setting the homeostatic conditions, reverting potentially detrimental trends, and removal of potentially toxic intermediates (Fig. 2c; see also refs 20 and 23).

### Alterations of gene expression linked to entry into quiescence

**Figure 3:**
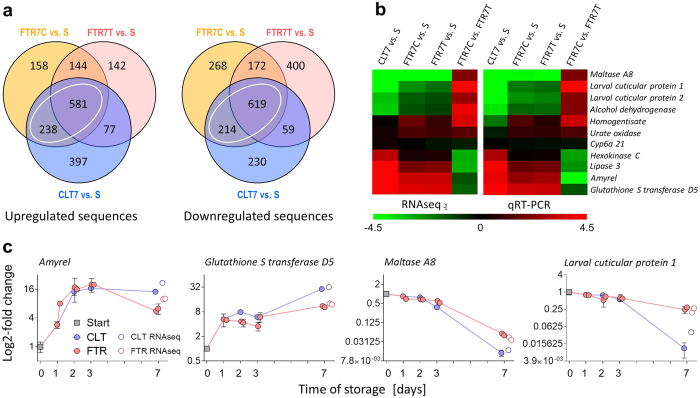
Cold-induced changes in transcriptome identified using RNAseq. (**a**) Venn’s diagrams showing the numbers of significantly (log2-fold change > ± 0.55, *q* value < 0.05) up- and down-regulated mRNA transcripts in quiescent larvae of *Drosophila melanogaster* stored at CLT 6 °C and FTR 6 °C/11 °C for 7 days compared to Start of the experiment (S). For complete dataset, see Table S2, parts A–F. White ovals in Venn’s diagrams show cold-responding differentially expressed sequences shared between CLT and FTR that were later subjected to enrichment analysis (Table S3, parts A–F). (**b**) the reliability of RNAseq results was validated using qRT-PCR analysis of mRNA abundances in 11 selected sequences relative to two reference sequences, *Ribosomal protein L32* and *beta Tubulin 56D* (Table S2, part G). Heat maps code for log2-fold changes in RNAseq and qRT-PCR analyses of the same total RNA samples. (**c**) examples of cold-induced changes in four sequences identified in the extended validation of RNAseq results using qRT-PCR. Additional samples of total RNA were collected on days 1, 2, and 3 of storage at CLT and FTR to describe the dynamics of transcriptomic changes during cold storage.

Gene expression patterns changed profoundly in response to cold exposure irrespective of whether it was applied as the FTR or CLT regime. All details on identity of differentially expressed (DE) sequences, including exact log2-fold changes and the results of statistical analysis, are summarized in Table S2, parts A–F. The Fig. 3a shows that 1,737 and 1,962 sequences were significantly up- and down-regulated, respectively, when comparing the transcriptomes analysed on day 7 of cold exposure to the Start of experiment (three different end points: CLT7 and FTR7 sampled at the end of the cold [C] and warm [T] episode *vs.* single start point [S]). Direct validation using qRT-PCR analysis in 11 selected genes confirmed high reliability of our RNAseq results (Fig. 3b).

The Venn’s diagrams in Fig. 3a suggested that a large part of the gene expression response to cold was common to FTR and CLT regimes. White ovals delimit numbers of cold-responding DE-genes shared between FTR and CLT conditions: 819 up-regulated and 833 down-regulated in total. We performed the enrichment analysis of these shared DE sequences (Table S3, parts A, B) and found that the gene categories associated with catabolic processes of carbohydrates, lipids, and amino acids, respiration, and energy production were significantly under-represented. Diverse glycosyl hydrolases, and among them specifically mannosidases, formed the most down-regulated category. Mannosidases are enzymes degrading yeast cell walls containing polymannose chains^34^. These results indicated, in agreement with metabolomics, that digestion of food, catabolism, and energy production were not only severely compromised, but probably also actively down-regulated (as indicated by relatively low abundances of relevant transcripts) upon transfer to low temperatures. Conversely, the gene categories associated with activity of glutathione transferases and metabolism of xenobiotics were significantly over-represented under CLT and FTR conditions compared to Start. These results identify metabolic disorders (accumulation of potentially toxic intermediates), loss of redox balance, and oxidative damage as potential sources of indirect chill injury linked to storage at low temperatures (see also refs 27,35 and 36).

The global difference in gene expression between FTR and CLT regimes was relatively small. When comparing two treatments with the same end-temperature of 6 °C, *i.e.* FTR7C vs. CLT7, 295 sequences were up-regulated, whereas 217 sequences were down-regulated (see red ovals in the Venn’s diagrams associated with Table S3, parts C, D). The enrichment analysis of these DE-genes identified that some GO terms associated with chitin metabolism and cuticle formation were differently regulated between CLT and FTR. Such differences might be related to slow development in cuticle, the dynamism of which was probably suppressed more at CLT than at FTR (see our previous discussion on incomplete developmental arrest in quiescence). Other differences between CLT and FTR concerned the GO terms associated with either glycosyl hydrolases on one side or iron binding, redox reactions, and glutathione transferases on the other side, *i.e.* the processes that were generally either down- or up-regulated, respectively, under both CLT and FTR conditions (Table S3, parts C, D). To further document the overall similarity in expression trends under two thermal regimes, CLT and FTR, we performed an extended qRT-PCR validation analysis of mRNA transcripts in four selected genes, *maltase A8*, *glutathione transferase D5*, *larval cuticular protein 1,* and *amyrel*. Results of extended validation exemplify that relatively small differences in gene transcript abundances on day 7 might arise simply as a result of slightly different rates of generally similar trends under CLT and FTR conditions (Fig. 3c).

Only 116 sequences were up-regulated and 126 sequences were down-regulated in the T vs. C episode of FTR (Table S3, parts E, F). The effect of altering the warm and cold episodes during FTR was analysed using enrichment analysis of these DE-genes. No significantly warm-over-represented gene category was found. We found only one significantly warm-under-represented category that included four cysteine-rich genes with unknown function (described by two different terms: SM00689 and IPR006611). In addition, the category of stress genes, including several heat shock factors (*hsr omega* and *hsf*) and genes coding for proteins involved in response to extreme temperatures (*DnaJ*, *Hsp60*, *Hsc2*, and *Hsp83*), was found to be marginally significantly warm-under-represented (meaning: cold-over-represented).

### Differences in physiology between the larvae stored at CLT vs. FTR

**Figure 4:**
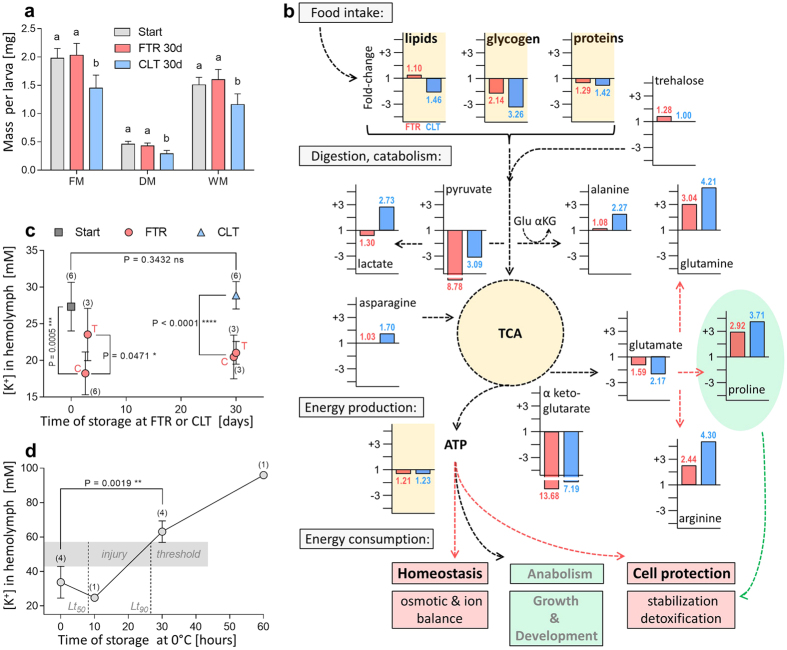
Changes in physiological parameters during 30-d-long storage at CLT and FTR. (**a**) Differences in fresh mass (FM), dry mass (DM), and water mass (WM) in quiescent larvae of *Drosophila melanogaster* stored at CLT 6 °C and FTR 6 °C/11 °C for 30 days compared to Start of the experiment. Differences were analysed using an ANOVA followed by Bonferroni’s post hoc test. (**b**) Schematic depiction of major processes, catabolic and energy production pathways, selected energy substrates, metabolites, and ATP. Metabolomic and transcriptomic analyses collectively suggest that processes of food intake, digestion, catabolism, and energy metabolism are generally suppressed during quiescence (dashed black arrows). The growth and development, and associated anabolic processes, are arrested. In contrast, the metabolic pathways resulting in accumulation of some amino acids, and the processes linked to homeostasis and cell protection are relatively bolstered in quiescent larvae (dashed red arrows). The small diagrams represent fold-differences in levels of selected compounds between larvae collected at the Start of experiment and after 30 days of storage at FTR (red columns and numbers) and CLT (blue columns and numbers). Some of the accumulated amino acids may stabilize cells of quiescent larvae (proline, dashed green arrow). For complete dataset, see Table S4. (**c**) Homeostasis of potassium concentration in haemolymph during 30-d-long quiescence at FTR and CLT (C and T, samples collected at the end of cold and warm episodes, respectively, of FTR; numbers within parentheses above data points represent total number of all individuals) Differences were analysed by Student’s *t*-test. (**d**) Rapid development of hyperkalaemia (failure of homeostasis) upon transfer of the 25 °C-acclimated larvae to 0 °C. The lethal times *Lt*_*50*_ and *Lt*_*90*_ for mortality of 50% and 90%, respectively, larvae exposed to 0 °C are taken from our earlier study^11^.

We compared the mass parameters, hydration, basic biochemical composition, selected metabolites, total ATP content, potassium concentration in haemolymph, and two biomarkers of oxidative damage in the larvae collected at the Start of the experiment and after 30 days of storage at CLT or FTR. We found that larvae experienced significant losses of fresh mass (FM, 26.8%), dry mass (DM, 37.0%), and water mass (WM, 23.2%) at CLT, which was in striking contrast to FTR-stored larvae that were able to maintain all these parameters practically constant (Fig. 4a, Table S4). The most plausible explanation for this difference was that the larvae stored at FTR ingested food during warm episodes, whereas the larvae stored at CLT were starving. Therefore, the CLT-stored larvae probably had to rely on their internal reserves much more than FTR larvae. Consequently, CLT-stored larvae exhibited massive depletions of energy substrates: 69.0% of glycogen, 31.3% of proteins, and 29.5% of lipids were lost within 30 days (Fig. 4b, Table S4). Extrapolating these decreasing trends to longer durations of storage, it is reasonable to expect that the depletion of energy substrates, especially of glycogen, will limit storage time at CLT at some point lying close to the empirically observed limit of 60 days. Interestingly, the FTR-stored larvae were able to maintain their lipid reserves constant for 30 days, similar to diapausing larvae of *C. costata* stored at 4 °C^7^. In holometabolous insects, the fat stores accumulated during the larval stage are brought forward to the pupa and serve as energy stores for metamorphosis and early adult life^37^. *D. melanogaster* pupae consumed 35% and 27% of their initial lipid and carbohydrate reserves, respectively, to fuel metamorphosis into the adult form^38^. Therefore, even a partial depletion of energy stores during the larval stage may critically limit survival during future ontogenetic steps.

The alteration of metabolic pathways during larval quiescence, either at CLT or FTR, resulted in depletion of specific intermediary metabolites (pyruvate, ketoglutarate, and glutamate), whereas other metabolites were accumulated (glutamine, proline, and arginine) (Fig. 4b, Table S4). Interestingly, we recently showed that all three accumulated metabolites could stimulate high freeze-tolerance in larvae of *D. melanogaster* when applied as food additives^39^. In theory, the accumulated proline and glutamine, probably in combination with trehalose and some other amino acids, could stabilize cells by preventing or reducing partial unfolding of proteins and membrane fusion in the larvae exposed to thermal stress linked to quiescence^40^,^41^. The cryoprotective function of arginine is probably exerted specifically during freeze dehydration, which is not relevant for storage in quiescence (for discussion on cryoprotective roles of metabolites, see ref. 39). Natural accumulation of proline has been observed in various insects during cold acclimation^42^,^43^,^44^. The cryoprotective role of proline was directly proven in plants^45^,^46^ and proline was found to be essential for cryopreservation of *C. costata* larvae in liquid nitrogen^7^. It seems likely that the cold-induced accumulation of proline, and its metabolic associates, has been bolstered in the process of evolutionary adaptation to cold in some cold-hardy organisms. No, or only a weak, evolutionary adaptation to cold, however, is expected in larvae of *D. melanogaster.* These larvae were designed by evolution for rapid growth and development under warm conditions of tropics^12^. Our results strongly support the view that the accumulation of proline is a widespread feature resulting from redirecting the metabolic pathways during metabolic suppression. As such, the accumulation of proline might serve as pre-adaptation in natural selection of organisms forced into dormancy during cooling of their habitats^47^.

The two metabolites that specifically accumulated only in CLT-stored larvae were lactate and alanine (Fig. 4b, Table S4). These results suggested that CLT-stored larvae had to rely more than FTR-stored larvae on ATP production via the anaerobic glycolytic pathway, and/or that CLT-larvae were not able to effectively metabolize the end products of the fermentation pathways. Despite this difference, the larvae were similarly balancing the processes of ATP production and consumption at both regimes, CLT and FTR. Relatively small and similar decreases of total ATP levels were observed during 30 days of storage at CLT (18.9% of ATP lost) and FTR (17.7% of ATP lost) (Fig. 4b, Table S4). The balanced supply of ATP is critically needed to secure basic metabolic and homeostatic processes during quiescence. For instance, the quiescent larvae proved their ability to maintain relatively low concentrations of potassium in their haemolymph for at least 30 days at both CLT and FTR conditions (Fig. 4c). In contrast, the characteristic increase of extracellular potassium concentration (hyperkalaemia) occurred in the larvae when we transferred them from 25 °C to 0 °C (Fig. 4d). Within 30-min of exposure at 0 °C, the potassium concentration significantly increased, almost 2-fold, compared to initial conditions and the insects died. Cold-induced hyperkalaemia was shown to be strongly correlated with the degree of chilling injury in different insects^14^,^15^,^48^,^49^. The cold-induced hyperkalaemia is believed to stem from an inability to energize the primary ion pumping systems in the hind gut and/or Malpighian tubules and, consequently, to maintain organismal osmotic and ionic homeostasis. Hyperkalaemia leads to cell membrane depolarization, which probably initiates a cascade of detrimental processes, including uncontrolled activation of cellular proteases and lipases that ultimately destroy cell integrity^13^,^50^,^51^.

Results of previous studies^27^,^35^,^36^ and those of our enrichment gene expression analysis (Table S3) have suggested that metabolic disorder and oxidative stress might be important causes of indirect chilling injury. Therefore, we assessed the levels of lipid hydroxyperoxides (LPOs) and protein carbonyls (PLs) in the quiescent larvae stored at CLT and FTR. Indeed, we found that CLT-stored larvae might suffer from oxidative stress as the levels of both biomarkers of oxidative damage were slightly elevated. Although these elevations were only marginally significant in statistical terms, they may represent actual tolerable limits, as suggested by a parallel analysis of stress biomarkers in control larvae exposed to paraquat-augmented diet (for more details, see Table S4). No elevations of LPO and PL levels were observed under FTR conditions. These results suggest that while the detoxification and anti-oxidant systems were transcriptionally bolstered in quiescent larvae at both CLT and FTR conditions, the systems were functioning more efficiently at FTR than at CLT conditions.

## Methods

### Insects, thermal conditions and survival/storability assays

All experiments were conducted with Oregon R strain of *Drosophila (Sophophora*) *melanogaster*^52^. The stock strain has been maintained for decades in our laboratory in glass tubes (12 cm high, 2.5 cm in diameter) at constant 18 °C with 12-h/12-h light/dark (L/D) cycle in incubators MIR 154 (Panasonic Healthcare, Gunma, Japan). Each tube contained 5–10 g of artificial diet composed of agar (1%), sugar (5%), yeast (4%), cornmeal (8%), and methylparaben (0.2%). In order to obtain synchronously developing cohorts of larvae for experiments, approximately 50 pairs of flies (25 females) were allowed to lay eggs for 24 h at the conditions specified above. Next day, embryos were transferred to constant 15 °C with 12-h/12-h light/dark (L/D) cycle and reared until the first wandering larvae occurred, typically on day12 of larval age. Next, all wandering larvae were removed and the tubes with remaining larvae were transferred to constant darkness and experimental thermal regimes. Using the larval stage, rather than adult stage, of *D. melanogaster* for experiments was based on our earlier experience with this model. In previous papers, we compared cold- and freeze-tolerance in the larvae of two drosophilid flies, sub-arctic diapausing *C. costata*^7^ and tropical non-diapausing *D. melanogaster*^8^,^11^.

We have assayed survival and storability of larvae exposed to different thermal regimes for different periods of time ranging from 1 d to 60 d. Five constant low temperatures (CLTs: 3 °C, 5 °C, 6 °C, 7 °C, 9 °C) and two fluctuating thermal regimes [FTRs: 20-h of 5 °C/4-h of 11 °C (in brief: FTR 5 °C/11 °C) and 20-h of 6 °C/4-h of 11 °C (in brief: FTR 6 °C/11 °C)] were tested (Fig. S1a). The thermal conditions were set in incubators MIR 154. The random fluctuations around the set point were not exceeding ± 0.2 °C, which was verified by direct temperature recordings inside the larval diet using the datalogger S0122 equipped with external Pt1000 temperature probe (Comet System, Roznov pod Radhostem, Czech Republic). The chosen thermal conditions for experiments were based on: (i) literature data showing that the lower developmental threshold temperature for larval and pupal development is 10–11 °C (Loeb and Northrop, 1917; Bliss, 1927); (ii) our earlier observations showing that temperatures below a threshold of 6 °C rapidly caused mortality^11^; and (iii) our preliminary assays showing that higher temperatures of warm period (12 °C) and/or longer warm periods (6 h, 12 h at 11 °C) allow most larvae continuing into wandering stage and making unsuccessful attempts to pupariate (called ‘pupariation mortality’ in this study) within 2–4 weeks.

After the exposure to given thermal conditions, tubes containing insects were moved to room temperature (at the end of warm period) and allowed to equilibrate for 1 h. Then, all insects were inspected under binocular microscope (larvae still inside diet were washed out) and scored to three categories: live larva in the diet or on glass wall (classified as ‘larva’), dead larva in the diet (counted to total *n* but not classified), and malformed puparium on glass wall (classified as ‘pupariation mortality). All live larvae were moved to fresh diet and kept for another 30 d at constant 18 °C with L12/D12 photoperiod to check their ability to continue development. Pupariation and eclosion of fit adults were scored as ultimate survival criterions. Based on results of survival assays, two regimes were selected: CLT 6 °C and FTR 6 °C/11 °C (Fig. S1a) and subjected to more detailed metabolomic, transcriptomic and physiological analyses. In order to sample only living larvae for analyses, we observed the larvae under binocular microscope for short time (up to 5 min) after washing them out of diet using cold water (11 °C) and selected only those specimens showing spontaneous movements. The general abbreviations CLT and FTR will mean CLT 6 °C and FTR 6 °C/11 °C regimes, respectively, in the following text unless specified otherwise.

### Metabolomics

Larvae were sampled after 0, 1, 2, 3 and 7 d- exposures to thermal regimes CLT vs. FTR. In case of FTR, larvae were sampled twice a day, first time at the end of cold period (6 °C, labelled as C) and second time at the end of warm period (11 °C, labelled as T) (see Fig. S1a). Whole larvae (pools of 4 larvae in 6 replications) were homogenized twice in 400 μl of 70% ethanol and the extracts were subjected to complex analysis of major metabolites using a combination of mass spectrometry-based analytical methods as described earlier^11^. Low-molecular-weight sugars and polyols were determined after *o*-methyloxime trimethylsilyl derivatization using gas chromatograph (GC) with flame ionization detector GC-FID-2014 equipped with AOC-20i autosampler (both from Shimadzu Corporation, Kyoto, Japan). Profiling of acidic metabolites was done after the treatment with ethyl chloroformate under pyridine catalysis and simultaneous extraction in chloroform^53^ using Trace 1300 GC combined with single quadrupole mass spectrometry (ISQ-MS) (both from Thermo Fisher Scientific, San Jose, CA, USA) and liquid chromatograph Accela LTQ XL with linear ion trap combined with high resolution mass spectrometers Q Exactive Plus coupled with Dionex Ultimate 3000 (all from Thermo Fisher Scientific). The metabolites were identified against relevant standards and subjected to quantitative analysis by using an internal standard calibration method. All standards used were purchased from Sigma-Aldrich (Saint Luis, MI, USA).

Metabolite profiles were analysed using a between-class principal component analysis (PCA)^54^ to test clustering effects according to the experimental modalities. The between-class PCA focuses on differences among the classes defined as qualitative instrumental variables. A Monte-Carlo test (number of iterations = 1,000) was used to determine whether the samples were randomly distributed in variable space according to their experimental modality. All data were scaled and mean-centered prior to the PCA. All analyses were performed using the ade4 library in the statistical software R 3.0.3 (R Development Core Team, Vienna, Austria).

### RNAseq and gene expression enrichment analysis

Whole larvae were sampled (pools of 8 larvae in 3 replications) at the start of experiment (Start) and after 7 d-long exposure to thermal regimes CLT vs. FTR and subjected to transcriptomic analysis. The total RNA was extracted using the RiboZol RNA Extraction Reagent (Amresco, Solon, OH, USA). Pellet of total RNA was dissolved in 20 μl of DEPC-treated water and an aliquot of 5 ul was taken for total RNA quality assessment on denaturing agarose gel and concentration measurement using NanoDrop 2000 (ThermoFisher Scientific, Waltham, MA, USA). The total RNA concentrations were levelled exactly to 0.5 ug/ul and the samples were either sent to the EMBLGenomics Core Facilities (GeneCore, Heidelberg, Germany) for cDNA library production and Illumina RNAseq or used for direct qRT-PCR validation of RNAseq results. The cDNA libraries were prepared using Covaris S2 (Covaris, Woburn, Massachusetts, USA) for fragmentation aiming for an insert size of about 150 nt and TruSeq RNA sample prep kit (Illumina, San Diego, California, USA). The cDNA libraries were then sequenced using 50 nt single end sequencing on HiSeq2000 sequencer (Illumina, San Diego, California, USA).

The quality of RNAseq results was first assessed using FastQC (http://www.bioinformatics.babraham.ac.uk/projects/fastqc/). The raw reads were trimmed and all adapters and overrepresented sequences were removed with Trimmomatic software^55^. The resulting reads were filtered with a Phred quality score of at least 28. Reads were then mapped to *Drosophila melanogaster* genome (dm3) using TopHat 2^56^ with default parameters. Aligned reads were then assembled into transcripts defined by coordinates using Cufflink (-I 300000 -F 0.1 -j 0.15 -p 6)^57^. Finally, the transcript differential expressions were calculated using Cuffdiff (geometric normalization, pooled dispersion estimation, 10 minimum alignment count, and cufflinks effective length was applied)^58^. Transcripts were considered significantly differentially expressed (DE) when the Benjamini-Hochberg-corrected *p*-value (*q*-value) was below 0.05 and the absolute log2-fold change was above 0.55. To detect if any particular ontology was enriched in our comparisons, we conducted an enrichment analysis using David software^59^. Gene categories or gene classification terms [GO term, InterPro classification, KEGG ID, Cluster of Orthologous Genes (COG), PIR superfamily, SMART accession, etc.] were considered significantly enriched when a corrected Bonferroni *p*-value was below 0.05.

### qRT-PCR validation of RNAseq results

The aliquots of total RNA that was subjected to the RNAseq experiment were taken for direct validation of the RNAseq results. The 5 uL (2.5 ug) aliquots of total RNA were treated with DNase I (Ambion, Life Technologies) followed by the first strand cDNA synthesis using Superscript III (Invitrogen, Carlsbad, CA, USA). The cDNA products (20 μL) were diluted 25 times with sterile water. Relative abundances of mRNA transcripts for selected transcripts were measured by quantitative real time PCR (qRT-PCR) using the CFX96 PCR light cycler (BioRad, Philadelphia, PA, USA) and the LA Hot Start Master Mix (Top-Bio, Vestec, Czech Republic). PCR reactions were primed with a pair of oligonucleotide primers specific for 11 selected genes (Table S2, part G). Emission of a fluorescent signal resulting from SYBR Green binding to double-stranded DNA PCR products was detected with increasing PCR cycle number. Quantitation cycle (*C*_Q_) for each sample was automatically calculated using the algorithm built in the CFX96 PCR light cycler software. The levels of mRNA transcripts of *Ribosomal protein L32 (Rpl32*) and *beta-tubulin 56D (β-tub*) served as endogenous reference standards for relative quantification of the target transcript levels (See Figure associated with Table S2, part G and^60^). Each sample was run as a doublet (two technical replicates) of which the mean was taken for calculation. Relative ratios of the candidate mRNA levels (*C*_Q_) to geometric mean of the levels (*C*_Q_) of two reference gene mRNAs were calculated according to^61^.

Next, we performed an extended validation of our RNAseq results using qRT-PCR of four selected genes: *Amyrel*, *Gst D5*, *Maltase A8*, and *Lcp 1*. For this validation, we added total RNA extracted from larvae that were sampled on days 1, 2 and 3 of storage at CLT or FTR regimes. The processing of larval samples to cDNA was equal as described above.

### Physiology

We sampled the larvae at the start of experiment (Start, S) and after 30 d-long exposure to thermal regimes CLT vs. FTR (the FTR sample was taken at the end of cold period). Fresh mass (FM) was measured individually in 20 larvae in each treatment using Sartorius balance with sensitivity of 0.01 mg. Dry mass (DM) was measured after drying the specimens at 65 °C for 3 days. Water mass (WM) and hydration were calculated from gravimetric data.

Total water soluble proteins were measured in a sample pooled of 5 larvae (3 replications for each treatment) by the bicinchoninic acid protein assay^62^ after extraction in 50 mM Tris, pH 7.2. Glycogen was extracted from a sample pooled of 5 larvae (3 replications) in hot alkali^63^ from the pellet remaining after removal of simple sugars using homogenization twice in 400 μl of 70% ethanol and centrifuging at 20,000 g/4 °C/10 min. Glycogen was assayed using the colorimetric determination with phenol and concentrated sulphuric acid^64^. Total lipids were measured in a sample pooled of 5 larvae (3 replications) using spectrophotometric analysis with phosphoric acid-vanillin solution^65^ after extraction of lipids by using chloroform:methanol solution (2/1, *v/v*)^66^. Total ATP concentration in muscle tissue dissected from a pool of 10 larvae (3 to 6 replications) was measured using enzymatic assay of luciferase that consumes ATP for convertion of beetle luciferin to oxyluciferin (CellTiter-Glo Luminescent Cell Viability Assay, Promega, Madison, WI, USA).

Based on results of metabolomic analysis, we selected 10 most important metabolites (pyruvate, lactate, ketoglutarate, arginine, glutamine, aspargine, alpha alanine, proline, glutamate and trehalose, together representing more than 75% of the total metabolite pool on day 7) and conducted their targeted quantification using methods described above. Pools of 5 larvae were sampled in 3 biological replicates for each treatment.

The concentration of potassium ions was measured using MI-442 K^+^ Ion Microelectrode in combination with reference electrode MI-402 (both from Microelectrodes Inc., Bedford, NH, USA). A sample of hemolymph was collected from a pool of 10–20 larvae (to reach ca. 3 μl in total) into calibrated micro-capillary tube (Broomall, PA, USA). Exactly 2.5 μl of hemolymph was then diluted 3 times with 5 μl of deionized water in order to obtain sufficient volume for microelectrodes (7.5 μl). Three to six biological replicates (pools of 10–20 larvae) were measured in each treatment. Manipulation with 10–20 larvae (removal out of diet, tearing, collecting the hemolymph into capillary) took approximately 2–3 min, during which the larvae were maintained at the same temperature as in the treatment (Start, 15 °C; CLT, 6 °C; FTRC, 6 °C; FTRT, 11 °C). Voltage was measured using pH/mV Hand-Held Meter pH 330 (WTW, Weilheim, Germany) and converted to [K^+^] using semilog line regression calibration curve. The calibration samples (1 mM, 10 mM, 100 mM KCl solution) were measured just prior to measuring the samples on every occasion.

The levels of two biomarkers of oxidative stress were analyzed in larval muscle tissue dissected from 10 larvae in three biological replicates for each treatment. Lipid hydroxyperoxides (LPOs) were quantified using colorimetric assay utilizing redox reaction of LPOs with ferrous ions (LPO Kit ab133085, Abcam, Cambridge, UK). The amount of LPO was expressed as percentage of total phospholipids extracted from larval muscle in chloroform:methanol (2:1, v/v) followed by acetonitrile/hexane phase separation as described earlier^67^. Protein carbonyls (PCs) were analyzed after their tagging with DNPH followed by colorimetric assay of DNP hydrazones formed in the reaction provided in the Protein Carbonyl Assay Kit (ab126287, Abcam). PCs were expressed in nmols per total protein extracted from larval muscle as described above.

## Additional Information

**How to cite this article**: Koštál, V. *et al.* Physiological basis for low-temperature survival and storage of quiescent larvae of the fruit fly *Drosophila melanogaster. Sci. Rep.* **6**, 32346; doi: 10.1038/srep32346 (2016).

## Supplementary Material

Supplementary Information

Supplementary Table S1

Supplementary Table S2

Supplementary Table S3

Supplementary Table S4
