# Supplementary material for: Physiological basis for low-temperature survival and storage of quiescent larvae of the fruit fly Drosophila melanogaster
Source: Sci Rep. 2016 Aug 30;6:32346. doi: 10.1038/srep32346 (PMC5004108; doi:10.1038/srep32346)
Supplement: Supplementary Information [file srep32346-s1.doc]

**Physiological basis for low-temperature survival and storage of quiescent larvae of the fruit fly *Drosophila melanogaster***

Vladimír Koštál, Jaroslava Korbelová, Tomáš Štětina, Rodolphe Poupardin, Hervé Colinet, Helena Zahradníčková, Iva Opekarová, Martin Moos & Petr Šimek

**Supplementary information**

**
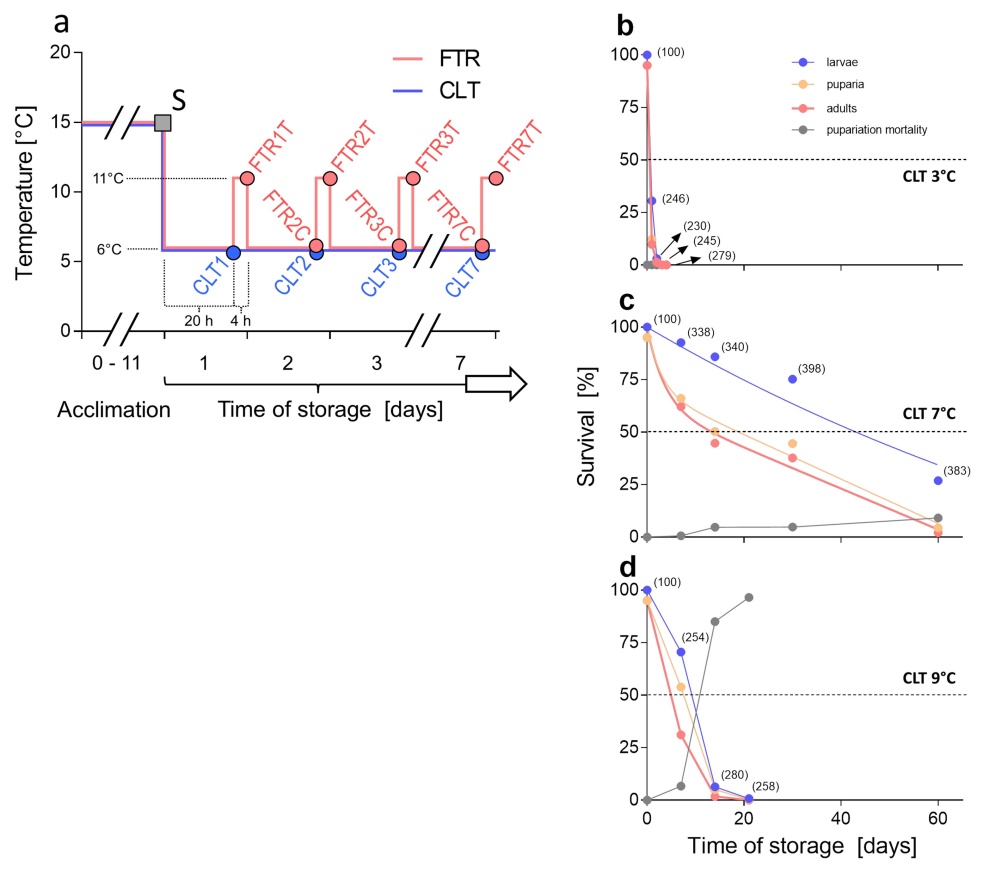
**

**Figure S1**

**Thermal regimes CLT and FTR and survival under selected CLT regimes.**

(**a**) Schematic depiction of two thermal regimes, constant low temperature (CLT) and fluctuating thermal regime (FTR) used to store 3rd instar larvae of *Drosophila melanogaster* in a state of low temperature quiescence. The samples of larvae for assessment of survival and for different analyses were taken at the Start of experiment (S, grey square) and after storage at CLT or FTR regime for certain time (blue and red circles, respectively). (**b-d**) Survival of larvae under three different CLTs of 3°C (**b**), 7°C (**c**), and 9°C (**d**). Three different levels of survival were scored: live larvae (blue circles, larvae showing spontaneous movements); puparia (orange circles, formation of morphologically normal puparium); and adults (red circles, eclosion of morphologically normal adult). In addition, we counted numbers of malformed puparia on the wall of the glass tube (grey circles) and scored them as ‘pupariation mortality.’ Numbers within parentheses are the total number of all individuals (*n*, including dead larvae) recovered at each time. Two-phase exponential decay curves (Prism 6.0, GraphPad, San Diego, CA, USA) were fit to survival data at CLT 7°C (goodness of fit, R^2^, 0.9370, larvae; 0.9782, puparia; 0.9842, adults).
